# The Costs of Carnivory

**DOI:** 10.1371/journal.pbio.0050022

**Published:** 2007-01-16

**Authors:** Chris Carbone, Amber Teacher, J. Marcus Rowcliffe

**Affiliations:** Institute of Zoology, Zoological Society of London, London, United Kingdom; Princeton University, United States of America

## Abstract

Mammalian carnivores fall into two broad dietary groups: smaller carnivores (<20 kg) that feed on very small prey (invertebrates and small vertebrates) and larger carnivores (>20 kg) that specialize in feeding on large vertebrates. We develop a model that predicts the mass-related energy budgets and limits of carnivore size within these groups. We show that the transition from small to large prey can be predicted by the maximization of net energy gain; larger carnivores achieve a higher net gain rate by concentrating on large prey. However, because it requires more energy to pursue and subdue large prey, this leads to a 2-fold step increase in energy expenditure, as well as increased intake. Across all species, energy expenditure and intake both follow a three-fourths scaling with body mass. However, when each dietary group is considered individually they both display a shallower scaling. This suggests that carnivores at the upper limits of each group are constrained by intake and adopt energy conserving strategies to counter this. Given predictions of expenditure and estimates of intake, we predict a maximum carnivore mass of approximately a ton, consistent with the largest extinct species. Our approach provides a framework for understanding carnivore energetics, size, and extinction dynamics.

## Author Summary

Carnivores fall into two dietary groups based on the energetic requirements of their feeding strategies: small-bodied species, which feed mostly on prey smaller than themselves, and large-bodied species, which prefer prey around their own size. While carnivores around the size of a lynx or larger can obtain higher net energy intake by switching to relatively large prey, the difficulty of catching and subduing these animals means that a large-prey specialist would expend twice as much energy as a small-prey specialist of equivalent body size. Analyzing the balance between energy intake and expenditure across a range of species, we predict that mammalian carnivores should have a maximum body mass of one ton. Thus, mammalian carnivores are relatively small compared with the largest extinct terrestrial herbivorous mammals, such as the Indricothere, which weighed around 15 tons. The largest existing carnivore, the polar bear, is only around half a ton, while the largest known extinct carnivores, such as the short-faced bear, weighed around one ton. This study suggests that those extremely large carnivores would have been heavily reliant on abundant large prey, helping to explain why the largest modern mammalian carnivores are rare and vulnerable to extinction.

## Abstract 3

A simple theoretical model provides a framework to understand carnivore energy budgets and reveals insights into the evolution of body size in mammalian carnivores.

## Introduction

Predators face severe energetic constraints that affect many aspects of their ecology and evolution [1–4]. Many species in the Order Carnivora, for example, are at a high trophic level, with their population biomass representing only a small fraction of that of their prey [5]. The largest prey species themselves, can be unpredictable in space and time, widely dispersed, and rare [6–9]. Consequently, carnivores are some of the widest ranging terrestrial mammals for their size [6–8,10], and this affects overall energy budgets [11,12]. Carnivores also exhibit different hunting strategies in relation to their mass [3]. Smaller species (<15–20 kg) specialize in very small vertebrates and invertebrates which weigh a small fraction of their own weight [3,4]. The larger species (>15–20 kg), on the other hand, specialize in large-vertebrate prey near their own mass. Small-prey-feeding carnivores appear to have relatively low hunting costs—searching and pursuit can occur at walking pace and the capture and killing phases are often very brief [3,13–16]. Large carnivores, however, with their large prey, have higher hunting costs, with long high-speed chases and high costs of capture and killing [2,17–19]. Thus far, however, there has been no general framework to interpret adjustments in carnivore energy budgets associated with changes in body mass and hunting strategy. In this paper, we develop a simple model to examine adjustments in carnivore energetics in relation to predator and prey size and compare the model predictions with observed estimates of field metabolic rates (FMR) and intake rates. Our approach provides a framework to understand adjustments in carnivore energy budgets and provides insights into the evolution of body size in this diverse group.

## Results

In order to estimate the scaling of carnivore energy budgets, we develop a simple model that incorporates both the scaling of costs associated with body mass and the differences in time and energy budgets associated with hunting strategy. In our model, predatory carnivores are divided into two basic hunting groups (following [3]): those that feed on small prey (invertebrates <10 g and small vertebrates <2 kg) and those that feed on large prey (large vertebrates, typically >10 kg). We calculate daily energy expenditure (DEE) (kilojoules/day) as *E_r_T_r_* + *E_h_T_h_,* where *E_h_* and *E_r_* are the energetic expenditure rates while hunting and resting (kilojoules/hour) and *T_h_* and *T_r_* are time hunting and resting, respectively (hours/day). The scaling of the energetic costs of resting and hunting (in watts) was estimated from the allometric equations [2,20] *E_r_* = 5.5 *M* ^0.75^ and *E_h_* = 10.7 *M* ^0.684^ *v* + 6.03 *M* ^0.697^, where *M* is body mass in kilograms and *v* is the average speed (meters/second) during hunting [20] (see Materials and Methods). These equations allow us to estimate DEE, taking into account the effects of body mass and travel speed while hunting. We expect to see an increase in resting and hunting costs associated with an increase in size, but additionally, we expect a sharp rise in hunting costs as carnivores switch from feeding on small prey to large prey (see Materials and Methods).

**Figure 1:**
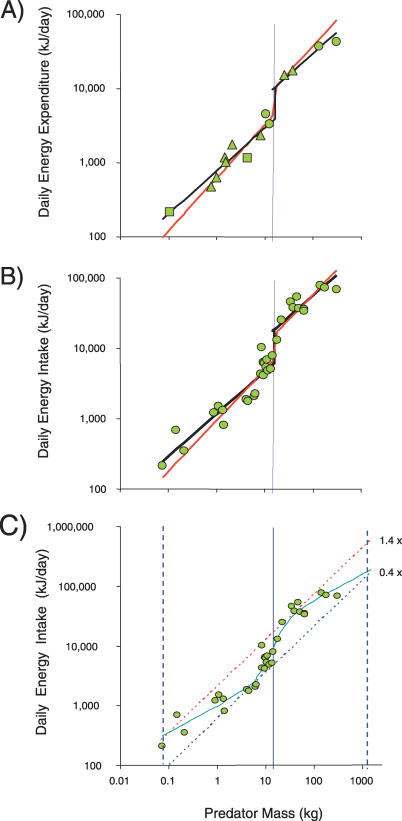
Estimates of DEE against Carnivore Mass (A) Estimates of DEE (kJ) against carnivore mass (kilograms) for 14 species, together with the model prediction of DEE (red line) and the piecewise regression fit (black line) (Table 1). Note the model predicts a step increase in DEE (by a factor of around 2.3) when carnivores switch to hunting large prey at the same mass where the piecewise regression breaks the data into two segments. Triangles represent estimates based on doubly labeled water, circles are based on behavioral observations, and squares are based on oxygen consumption in captive studies. The vertical line represents the predicted threshold of 14.5 kg where predators switch from small to large prey (see Figure 2). (B) Estimates of DEI (kJ) against carnivore mass (kilograms) for 32 species, together with the predicted DEI (red line) and piecewise regression fit (black line) (see text for details and Table 1)*.* (C) The scaling of DEI in relation to predicted DEI based on the scaling of FMR [12] with an exponent of 0.77, for upper (1.4 × predicted DEI, diagonal dashed red line) and lower (0.4 × predicted DEI, diagonal dashed blue line). These limits represent observed ranges of metabolic adjustments in energy budgets in carnivores. The sigmoid curve indicates the best-fit regression model (Table 1). The observed upper limit (1,100 kg) and lower limit (0.07 kg) of carnivore body masses are shown for comparison (blue vertical dashed lines), together with the predicted 14.5-kg threshold, separating small- from large-prey feeders (solid vertical line) (see text for details).

The model predictions for DEE (using parameter values in Materials and Methods) are compared against estimates of DEE for 14 species of free-ranging wild carnivores (Text S1) (Figure 1A). Given the higher travel speeds of the large-prey feeders, the model estimates an abrupt 2.3-fold increase in DEE with the switch to large-prey feeding. Consistent with this prediction, we find that a piecewise regression [21] provides a significantly better fit to the observed values than a simple linear regression (Table 1). This analysis, however, is limited by the number of direct estimates of DEE obtained in the field using doubly labeled water.

**Table 1:**
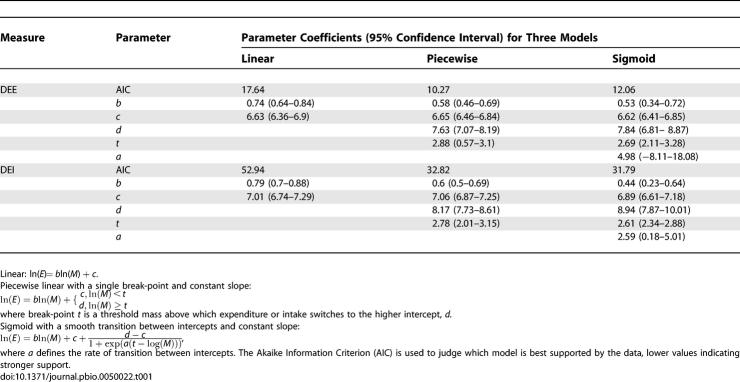
Three Alternative Models Were Fitted to Both DEE and DEI Values *(E)* in Relation to Species Body Mass *(M)*

**Figure 2:**
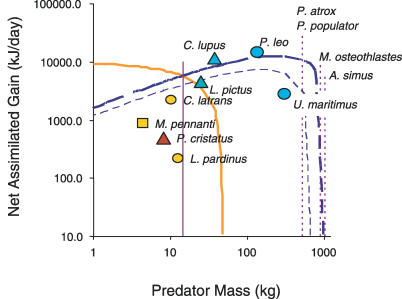
Net Rate of Assimilated Energy Gain Net rate of assimilated energy gain (kilojoules/day) showing predicted upper limits for the two dietary groups: small-prey feeders, (based on small vertebrates) (tan line) and large-prey feeders based on average intake (light blue dashed line) and maximum intake (dark blue dashed line). The net assimilated energy gain is calculated as 0.66 × DEI (kilojoules/day) − DEE [13]. The solid vertical line represents a 14.5-kg threshold where carnivores are predicted to switch from small- to large-prey feeding. A maximum mass of 700 kg or 1,100 kg is predicted for a large-vertebrate-feeding carnivore (based on the average and maximum coefficients of intake rate, respectively). Estimates for individual species above 4 kg are plotted with the model output. Triangles represent estimates based on doubly labeled water, circles are based on behavioral observations, and squares are based on oxygen consumption in captive studies. Fill color represents different diets: dark brown (invertebrates), light brown (small vertebrates), and blue (large vertebrates). The masses for four extinct species: Panthera atrox and P. populator (500 kg), Megistotherium osteothlastes (880 kg), and Arctodus simus (1,000 kg) are shown for comparison (broken vertical lines).

To provide a more robust test of the predicted increase in energy expenditure, we compared our model predictions against a surrogate for DEE, daily energy intake (DEI, kilojoules/day)—a measure that is more readily obtained in the field, and for which we found estimates for 32 species (see Materials and Methods, Text S1). As carnivores switch to hunting large prey, with a more than doubling of DEE, we expect a corresponding increase in DEI. We predicted DEI (kilojoules/day) from the model predictions of DEE, using the equation DEI = DEE/0.66. The constant was derived from an estimate of proportional assimilation efficiency for the value of animal food [13] (Figure 1B). Again, the observed pattern in DEI is consistent with the model predictions of DEI and we find a step change in observed DEI at the point where carnivores switch to large-prey feeding, with the piecewise regression model and a sigmoid regression providing significantly better fits than a simple linear regression of log intake rate against log body mass (see Materials and Methods) (Figure 1B).

Across all carnivores, the scaling exponents of DEE (0.74 ± 0.10, 95% confidence interval) and DEI (0.79 ± 0.09, 95% confidence interval) (Table 1) are similar to a three-fourths scaling expected based on metabolic theory [22] and the observed scaling exponent of FMR (used interchangeably with DEE) of 0.77 based on 58 eutherian mammals [12]. However, within small- and large-prey dietary groups, we see a secondary scaling pattern with DEE and DEI exhibiting a shallower scaling (0.58 ± 0.11 and 0.6 ± 0.09, respectively). In order to illustrate these primary and secondary scaling patterns in our surrogate for expenditure, DEI, we re-plot the data from Figure 1B with the best-fitting regression, the sigmoid model, together with trend lines based on the scaling exponent of 0.77 from FMR (converted to DEI) (Figure 1C). These trend lines illustrate upper and lower boundaries of 1.4 and 0.4 times estimated DEI, respectively, estimated from extremes in observed FMR in carnivores [2,13,23,24].

Within each dietary group, relatively small carnivores exhibit costly strategies (falling near the upper boundary); while relatively large carnivores exhibit energy conserving strategies (near the lower boundary). This suggests that within each dietary group, expenditure and intake rates impose increasing constraints as size within groups increases, leading to the shallower exponents within groups. The sigmoid curve fit of observed values of DEI intersects the upper and lower boundary of predicted DEI at masses near the size limits of the two dietary groups. At the upper size range of the large-vertebrate-prey feeders, the sigmoid curve intersects the lower boundary just above 1,100 kg, near the maximum mass estimated for some extinct carnivores (see below). At the lower end of the small-prey feeders, the sigmoid curve intersects at around 100 grams near the mass of the smallest carnivore species. The qualitative trend suggests that these upper and lower limits to DEE and DEI represent constraints on behavioral and metabolic adjustments, which then impose constraints on carnivore size.

In order to estimate net rate of gain (kilojoules/day) for these two dietary groups, the model predictions of DEE were combined with observed estimates of intake rate. Net gain *G* (kilojoules/day) was calculated as 0.66*IT_h_* − *E_r_T_r_* − *E_h_T_h_,* where *I* is intake rate per hour hunting (kilojoules/hour) and 0.66 is the assimilation coefficient (described above). We estimated the maximum sustainable mass for each dietary group by approximating the mass where *G* approached zero. Maximum intake rates for small-prey feeders were estimated as 746 and 1,496 kJ/h for invertebrates and small vertebrates, respectively (see Materials and Methods). For large-vertebrate-prey feeders, prey size increases with predator size [3,4,25], so we assumed that intake rate (kilojoules/hour) scaled with body mass^0.6^, with an intercept of 1,010 kJ/h at 1 kg body mass based on the observed scaling of DEI (converted to an hourly rate, Table 1) estimated from the above piecewise regression. We also used a maximum intercept of 3,132 kJ/h based on two well-studied large vertebrate hunting species (African wild dog Lycaon pictus and gray wolf Canis lupus) with very high rates of intake (see Materials and Methods). The model predictions in Figure 2 represent the upper limits of carnivore mass for the two dietary groups, and this is shown against observed estimates of net gain for several carnivore species for which we found independent estimates of DEE and DEI (Figure 2).

Using the above calculations, we predict a maximum carnivore mass of 18 or 45 kg (for invertebrate and small-vertebrate-prey feeders, respectively, Figure 2). Using estimates of intake rate for large-vertebrate prey, we predict a maximum carnivore mass of approximately 700 kg or 1,100 kg (for the average and maximum intercepts, respectively). These size limits are shown against the estimated masses of some of the largest known extinct mammalian carnivores. While we estimate that small prey can sustain carnivores up to 18–45 kg, above 14.5 kg carnivores feeding on large prey achieve a higher net gain.

## Discussion

Our model provides insights into dietary changes and the evolution of body size in mammalian carnivores. Using a simple energetic model, we predicted that hunting costs and resulting energy requirements would increase with an increase in prey size. We therefore expected to find a marked increase in DEE and DEI around the 14.5- to 21-kg size range, where carnivores are found to switch to hunting large-vertebrate prey [3]. This prediction was supported by the observed patterns in DEE and DEI (Figure 1). Our model also predicts that the transition between diet types in relation to predator mass may be predicted through the maximization of net gain rate. As body mass increases above 14.5–21 kg, the achievable net gain rate can be increased by adopting a large-prey feeding strategy. At this point, a switch to large prey leads to a doubling of DEE with a larger relative increase in DEI. The intake rates obtained from small prey are far lower than for large prey, and carnivores above 45 kg are predicted to be too large to sustain themselves on these types of prey [3]. The model also makes a less intuitive prediction that hunting large prey is less profitable for small carnivores (below 14.5 kg), because of the costs of hunting such prey are high relative to estimated intake rates. Previously [3], the rarity of smaller carnivores specializing in prey of their own size was noted but never explained. While the 14.5-kg threshold identified here is lower than the earlier estimate of 21 kg [3], it is within the range predicted by the earlier model (5–21 kg) and is within the confidence limits of the break points in our piecewise regressions of DEE and DEI (Table 1).

In previous research [3], patterns in prey selection with carnivore size were found to be similar across diverse species of carnivores. It is especially interesting to compare Felidae with Canidae—two families with a broad enough size range to span either side of the 15- to 20-kg threshold, but with very different ecologies. Canidae tend to be more social and employ a cursorial hunting strategy, while Felidae are likely to be solitary and stalking predators [4]. There are differing views about the potential benefits of social hunting [26–29] with a suggestion that it may be more beneficial to the Canidae, but despite the ecological, morphological, and taxonomic differences between these two groups, the patterns in prey size selection [3,4] and energy budgets, in relation to body size, appear broadly similar. It remains to be seen to what extent other aspects of carnivore ecology and evolution are affected by prey characteristics and adjustments in energy budgets, but this study presents an interesting framework to develop future research.

Although the primary scaling of DEE and DEI corresponds with the expected three-fourths power scaling of metabolic rate [12,22], a secondary shallower scaling of these variables occurs within dietary groups. This suggests that carnivores adopt behavioral and ecological strategies to minimize costs as their size approaches the limits imposed by intake rates and hunting costs linked with the size of their prey. The model predicts a discrete shift in prey size selected at 14.5 kg. However, in reality, there are a number of carnivores within the transitional range 15–20 kg, such as jackals and lynxes, which can effectively switch between hunting small prey and relatively large prey [3,4].

Our model assumes that the costs of transport scale with body mass according to typical mammalian estimates [20]. However, while medium to large carnivores exhibit transport costs consistent with other mammals [20,30], studies on the costs of locomotion for the very largest species, the lion Panthera leo [31] and polar bear Ursus maritimes [32], found that the costs were 2–3 times higher than expected for mammals of a similar size. These high costs of transport in the very largest carnivores may limit the ability to counter shortfalls in prey abundance simply by increasing hunting effort. How then do very large carnivores manage to balance energy budgets? Behavioral adjustments to reduce expenditure appear to be one important way in which this is achieved. For example, lions spend over 90% of their time inactive [33] and polar bears hibernate for short periods to reduce costs [24], while both species adopt hunting tactics that minimize time in active pursuit. But there must be physiological limits to the reduction of expenditure for an endothermic mammal.

In addition to the need to reduce expenditure, we predict some of the very largest extinct carnivores would consume about four times the intake estimated for lions [33]. Thus, these species are faced with two opposing constraints—the need to minimize expenditure while obtaining high rates of food intake.

Given these energetic constraints, we predict a maximum mass for a mammalian carnivore at 1,100 kg. Among extant species, the polar bear is the largest carnivore with the largest recorded individual weighing 1,002 kg [34]. However, at least four carnivore species with a very large average body mass are known to occur in the fossil record: the short-faced bear [35] *Arctodus simus,* estimated at 800–1,000 kg; the North American lion Panthera atrox [36] and the South American sabercat Smilodon populator [37,38], both around 500 kg; and Megistotherium osteothlastes (880 kg) [39]. While there is conflicting evidence about whether some of these species were pure carnivores (e.g., short-faced bear [40,41]), ancient bears had morphological similarities to the carnivorous polar bear. Thus, we believe that these species represent a possible upper limit to mammalian carnivore body size [1,42] and this provides support for our model prediction. This prediction, however, is based on mammalian metabolic rates and costs of transport. Were we to assume lower metabolic rates (e.g., as in reptiles), we would predict a larger maximum mass. The largest terrestrial predators, such as Giganotosaurus and Tyrannosaurs, may have achieved their massive size by having a lower metabolic rate, and it is interesting, in this context, that estimates of total metabolic rate for these species are similar to those of a mammal weighing about a ton [43].

Our analysis provides a broad perspective on energy and time budgets in mammalian terrestrial carnivores and provides insights into carnivore conservation and evolution, helping us to understand the vulnerability of large carnivores to historical and future extinctions. Among extant carnivores, the largest species are particularly vulnerable to human threat processes [44,45] and have been shown to have higher rates of extinction in the fossil record than smaller species, even prior to the evolution of man [1,42]. At the upper limits of body mass, an extremely high-prey biomass would be required to both minimize energy expenditure and maintain high rates of energy intake. Slight environmental perturbations, anthropogenic or otherwise, leading to lower prey availability, could readily upset this energy balance.

## Materials and Methods

### Estimates of time budgets, hunting travel speeds, and intake rates.

There were consistent differences in energy and time budgets for small- and large-prey-hunting carnivores. Our aim here was to broadly characterize these differences in the model. For carnivores hunting small prey (invertebrate and small vertebrates), there was no consistent differences in travel speeds or time budget across prey types or by predator sizes, so we used fixed average for travel speed of 1.19 km/h (*v* = 0.33 m/s) [14,46,47] and for time hunting *T_h_,* 9.8 h [13,14,47]. For the large-prey feeders, estimating the costs of hunting was more difficult because many activities contribute to hunting costs, including search, pursuit, capture, and killing large prey [19]. For this reason, we focused our estimate on cursorial predators—where much of the costs are expressed in terms of running speeds. We obtained our estimates from two well-studied large-prey-hunting carnivores, the African wild dog [2] and the wolf [12,28]. African wild dogs have average travel speed *v* of 8.3 m/s (30 km/h) [48], but since they are known to have unusually high rates of energy expenditure, we lowered *v* to a fixed rate of 7.4 m/s (26.5 km/h) to calibrate the model predictions to represent the average DEE for African wild dog [2] and the gray wolf [12], the only two large and wild terrestrial carnivore species for which DEE has been measured using doubly labeled water. We base our estimate of foraging time *T_h_* of 3.5 h [2] on wild dogs, but a review of hunting behavior of large carnivores showed that hunting times in these species were generally short, between 1.9 and 3.5 h [18,49] and very different from the longer hunting times observed in small carnivores [3,13–16].

### Estimates of intake rate.

Our analysis of net gain uses a maximum estimate of intake rate of 746 kJ/h for invertebrate feeders [13] and 1,496 kJ/h for small vertebrate feeders [14]. This assumes that intake rates are limited by the size of the prey and that all predators can successfully hunt the full range of small prey available [3]. For large-prey feeding, we estimate intake in relation to carnivore mass using the common slope from the piecewise regression model (Table 1), where *I* = *cM* ^0.6^ *.* We then estimated the coefficient *c* based on both the average coefficient from the piecewise regression (converted to an hourly rate) (*c*_ave_ = 1,010 kJ/h) and a maximum rate (*c*_max_ = 3,132 kJ/h) for the same two species (the African wild dog and gray wolf), which have high estimated intake rates for their size.

### Data.

Most average daily intake rates (data and sources are listed in Text S1) were obtained from independent estimates calculated by authors as wet weight (kilograms); others were calculated indirectly from prey masses and the number of prey eaten per day, assuming that invertebrate and small-vertebrate prey are consumed in entirety, while only a proportion of large-vertebrate prey are consumed (65%–91% depending on their mass [50]). Intake rates of large-prey feeders, such as lions, may be estimated over 2–3 d, then averaged to give a daily rate. Data points from 15 species are averaged from two or more independent estimates while 19 are from single sources. Caloric conversions for different food types were taken from the literature (see Text S1).

## Supporting Information

Text S1 Appendix (113 KB DOC)

## Abbreviations

DEE: daily energy expenditure
DEI: daily energy intake
FMR: field metabolic rate
