# Supplementary material for: The Costs of Carnivory
Source: PLoS Biol. 2007 Jan 16;5(2):e22. doi: 10.1371/journal.pbio.0050022 (PMC1769424; doi:10.1371/journal.pbio.0050022)
Supplement: Supplementary file 1 [file pbio.0050022.sd001.doc]

Appendix 1

The table shows Daily Energy Intake (DEI) and Daily Energy Expenditure (DEE) data with the references used. Fifteen DEI data points are averaged from two or more independent estimates whilst nineteen are from single sources. Caloric conversions for food types were 7,436 kJ/kg for invertebrate prey ^2, 3^, 6,682 kJ/kg for small vertebrate prey and 10,050 kJ/kg for large vertebrate prey ^1^. Body masses corresponding to DEE were taken directly from the literature from which the DEE value came (see DEE references) whilst those corresponding to DEI were taken from the Mammal SuperTeam database ^4^. Average prey mass values were obtained from ^5^.

1.	Creel, S. Cooperative hunting and group size: assumptions and currencies. *Animal Behaviour*, **54**, 1319-1324 (1997).

2.	Lloyd, B. D., McQueen, S. M. An assessment of the probability of secondary poisoning of forest insectivores following an aerial 1080 possum control operation. *New Zealand Journal of Ecology*, **24(1)**, 47-56 (2000).

3.	Meehan, T. D., Lease, H. M., Wolf, B. O. Negative indirect effects of an avian insectivore on the fruit set of an insect-pollinated herb. *OIKOS*, **109**, 297-304 (2005).

4.	Cardillo, M., Mace, G. M., Jones, K. E., Bielby, J., Bininda-Emonds, O. R. P., Sechrest, W., Orme, C. D. L., Purvis, A. Multiple causes of high extinction risk in large mammal species. *Science*, **309**, 1239-41 (2005).

5.	Carbone, C., Mace, G.M., Roberts, S.C. & Macdonald, D.W. Energetic constraints on the diet of terrestrial carnivores. *Nature*, **402**: 286-288 (1999).

| **Scientific Name** | **Common Name** | **Mass for DEE (kg)** | **DEE (kJ)** | **Mass for DEI (kg)** | **Intake (kg/day)** | **DEI (kJ)** | **DEE References** | **DEI References** | **Average Prey Mass (kg)** |
| --- | --- | --- | --- | --- | --- | --- | --- | --- | --- |
| Mustela nivalis | Least Weasel | 0.11 | 215.55 | 0.07 | 0.03 | 212.04 | 28 | 6, 28 | 0.03 |
| Mustella frenata | Long-tailed weasel |  |  | 0.15 | 0.10 | 686.20 |  | 33 | 0.02 |
| Mustela erminae | Stoat |  |  | 0.21 | 0.05 | 349.96 |  | 6, 35 | 0.05 |
| Bassariscus sumichrasti | Ring-tailed cat | 0.75 | 472.00 |  |  |  | 42 |  | 0.03 |
| Mustela putorius | Polecat |  |  | 0.90 | 0.18 | 1200.85 |  | 6 | 0.10 |
| Vulpes cana | Blandford's fox | 0.97 | 642.00 |  |  |  | 42 |  | 0.03 |
| Mustela vison | American mink |  |  | 1.09 | 0.22 | 1517.67 |  | 4, 36, 6, 27 | 0.10 |
| Martes martes | Pine marten |  |  | 1.38 | 0.19 | 1301.04 |  | 6 | 0.05 |
| Mungos mungo | Banded mongoose |  |  | 1.44 | 0.11 | 809.25 |  | 17 | 0.01 |
| Vulpes macrotis | Kit fox | 1.48 | 1180.00 |  |  |  | 42 |  | 0.70 |
| Vulpes ruepellii | Ruppell's fox | 1.54 | 1014.65 |  |  |  | 43 |  |  |
| Vulpes velox | Swift fox | 2.10 | 1780.00 |  |  |  | 42 |  | 1.35 |
| Otocyon megalotis | Bat eared fox |  |  | 4.17 | 0.25 | 1844.00 |  | 6 | 0.001 |
| Martes pennanti | Fisher | 4.43 | 1155.40 |  |  |  | 46 |  | 0.78 |
| Vulpes vulpes | Red fox |  |  | 4.56 | 0.47 | 1771.70 |  | 6, 31 | 0.48 |
| Nyctereutes procyonoides | Raccoon dog |  |  | 6.07 | 0.30 | 2058.60 |  | 6 | 0.05 |
| Felis chaus | Jungle cat |  |  | 6.56 | 0.33 | 2275.30 |  | 25 | 0.93 |
| Proteles cristatus | Aardwolf | 8.16 | 2368.00 | 8.50 | 0.58 | 4316.54 | 14 | 14, 29 | 0.001 |
| Canis adustus | Side-striped jackal |  |  | 8.56 | 1.50 | 10293.00 |  | 36 | 0.78 |
| Meles meles | Badger |  |  | 9.37 | 0.60 | 4117.20 |  | 6 | 0.03 |
| Felis canadensis | Canadian lynx |  |  | 9.38 | 0.92 | 6313.04 |  | 13 | 1.40 |
| Canis aureus | Golden jackal |  |  | 9.88 | 0.89 | 6126.87 |  | 16, 25 | 0.17 |
| Leptailurus serval | Serval |  |  | 10.59 | 0.85 | 5836.13 |  | 18 | 0.05 |
| Felis caracal | Caracal |  |  | 11.10 | 1.00 | 6862.00 |  | 25 | 16.50 |
| Felis pardalis | Ocelot |  |  | 11.90 | 0.70 | 4786.25 |  | 15 | 1.82 |
| Lynx pardinus | Iberian lynx | 12.68 | 3312.65 |  |  |  |  |  | 1.50 |
| Canis latrans | Coyote | 10.40 | 4511.47 | 13.42 | 1.48 | 5144.32 | 45 | 40 | 41.06 |
| Canis simensis | Ethiopian wolf |  |  | 14.50 | 1.15 | 7863.85 |  | 19 | 0.19 |
| Lynx lynx | Eurasian lynx |  |  | 17.80 | 1.31 | 13118.27 |  | 6,7,20 | 20.00 |
| Lycaon pictus | African wild dog | 25.00 | 15291.75 | 25.00 | 3.05 | 25122.90 | 41 | 4, 5 | 33.75 |
| Canis lupus | Timber wolf |  |  | 35.24 | 4.55 | 45720.77 |  | 6,12, 32 | 81.33 |
| Canis lycaon | Eastern wolf | 37.30 | 17699.60 |  |  |  |  |  |  |
| Panthera pardus | Leopard |  |  | 39.17 | 3.78 | 37938.75 |  | 1 | 33.00 |
| Acinonyx jubatus | Cheetah |  |  | 46.27 | 5.33 | 53566.50 |  | 5, 11, 34 | 33.75 |
| Puma concolor | Puma |  |  | 51.51 | 3.70 | 37185.00 |  | 8, 20, 38 | 76.00 |
| Crocuta crocuta | Spotted hyaena |  |  | 64.95 | 3.51 | 35275.50 |  | 2, 3, 10 | 107.33 |
| Panthera onca | Jaguar |  |  | 65.00 | 3.40 | 34170.00 |  | 8, 26 | 40.70 |
| Panthera leo | Lion | 136.00 | 36723.78 | 145.00 | 8.50 | 77887.50 | 5 | 5 | 137.25 |
| Panthera tigris | Tiger |  |  | 177.25 | 7.12 | 71556.00 |  | 9, 21, 22, 23, 24 | 189.25 |
| Ursus maritimus | Polar bear | 306.40 | 42383.90 | 306.40 | 6.82 | 68506.00 | 39 | 39 | 127.50 |

Data References

1	Bailey, T. N.	*The African Leopard - Ecology and Behaviour of a Solitary Felid* (Columbia University Press, New York, 1993).

2	Kruuk, H. *The Spotted Hyaena - A Study of Predation and Social Behaviour* (University of Chicago Press, Chicago, 1972).

3	Henschel, J. R. , Tilson, R. L.	How much does a spotted hyaena eat? Perspective from the Namib desert. *African Journal of Ecology* **26**, 247 – 255 (1988).

4	Creel, S. , Creel, N. W. *The African Wild Dog - Behaviour, Ecology and Conservation* (Princeton Uni Press, Princeton, 2002).

5	Schaller, G. B.	*The Serengeti Lion - A Study of Predator Prey Relations* (University of Chicago Press, Chicago, 1972).

6	Jedrzejewska, B. , Jedrzejewski, W. *Predation in Vertebrate Communities - The Bialowieza Primeval Forest as a Case Study* (Springer, New York, 1998).

7	Jobin, A. , Molinari, P. , Breitenmoser, U. Prey spectrum, prey preference and consumption rates of eurasian lynx in the Swiss Jura mountains. *Acta Theriologica* **45**, 243-252 (2000).

8	Nunez, R. , Miller, B. , Lindzey, F. Food habits of jaguars and pumas in Jalisco Mexico. *Journal of Zoology* **252**, 373 – 379 (2000).

9	Chundawat, R. S. , Gogat, N. , Johnsingh, A. J. T. Tigers in Panna: preliminary results from an Indian tropical dry forest In: *Riding the Tiger* Seidensticker, J. , Christie, S. , Jackson, P. (Cambridge University Press, UK, 1999).

10	Mills, M. G. L. *Kalahari Hyaenas. The Comparative Behavioural Ecology of Two Species* (Unwin Hyman Ltd, London, 1990).

11	Caro, T. M. *Cheetahs of the Serengeti Plains*	(University of Chicago Press, Chicago, 1994).

12	Palm, D.	2001	Prey selection, kill and consumption rates of Moose by Wolves in central Sweden	www.de5stora.com/illustrationer/fil_20041123132522.pdf	PhD thesis

13	Hebblewhite, M. , Paquet, P. C. , Pletscher, D. H. , Lessard, R. B. , Callaghan, C. J. Development and application of a radio estimator to estimate wolf kill rates and variance in a multiple-prey system. *Wildlife Society Bulletin* **31**, 933-946 (2003).

14	Williams, J. B. , Anderson, M. D. , Richardson, P. R. K. Seasonal differences in field metabolism, water requirements, and foraging behavior of free-living aardwolves. *Ecology* **78**, 2588-2602 (1997).

15	Emmons, L. H. A field study of ocelots (Felis pardalis) in Peru. *Revue d’Ecologie (Terre et Vie)* **43**, 133-157 (1998).

16	Sarker, N. J. , Ameen, M. N.	1990 Food habits of jackals (Canis aureus)	*Bangladesh Journal of Zoology* **18**, 189-202 (1990).

17	Rood, J. P. Population dynamics and food habits of banded mongoose. *East African Wildlife Journal* **13**, 89-111 (1975).

18	Geertsema, A. A. Aspects of the ecology of the serval Leptailurus serval in the Ngorongoro Crater, Tanzania. *Netherlands Journal of Zoology* **35**, 527-610 (1985)

19	Sillero-Zubiri, C. , Gottelli, D. Diet and feeding behaviour of Ethiopian wolves (Canis simensis). *Journal of Mammalogy* **76**, 531-541 (1995).

20	Kitchener, A. *The Natural History of The Wild Cats* (A & C Black, London, 1991)

21	Sankhala, K. S. *Tiger! The Story of the Indian Tig*er (Simon and Schuster, New York, 1997)

22	Schaller, G. *The Deer and the Tiger* (University of Chicago Press, Chicago, 1969)

23	Sunquist, M. E. The social organization of tigers (Panthera tigris) in Royal Chitwan national Park, Nepal. *Smithsonian Contributions to Zoology* **336**, 1-98 (1981).

24	Tamang, K. M. Population characteristics of the tiger and its prey	PhD Thesis, Michigan State University, East Lansing, 1979

25	Mukherjee, S. , Goyal, S. P. , Johnsingh, A. J. T. , Leite Pitman, M. R. P. The importance of rodents in the diet of jungle cat (Felis chaus), caracal (Caracal caracal) and golden jackal (Canis aureus) in Sariska Tiger Reserve, Rajasthan, India. *Journal of Zoology, London* **262**, 405-411 (2004).

26	Novack, A. J.	2003	Impacts of subsistence hunting on the foraging ecology of jaguar and puma in the Maya biosphere reserve, Guatemala.	MSc Thesis, University of Florida, USA	7-11 (2003).

27	Ferreras, P. , MacDonald, D. W. The impact of American mink *Mustela viso*n on water birds in the upper Thames. *Journal of Applied Ecology* **36**, 701-708 (199

28	Moors, P. J.	1977	Studies in metabolism, food consumption and assimilation efficiency of a small carnivore, the weasel (Mustela nivalis L.) *Oecologia* **27**, 185-202 (1977).

29	Richardson, P. R. K. Food consumption and seasonal variation in the diet of the aardwolf Proteles cristatus in southern Africa. *Zeitschrift für* *Saugetierkunde* **52**, 308-325 (1987).

30	Macdonald, D. W. Predation on earthworms by terrestrial vertebrates. In: *Earthworm Ecology from Darwin to Vermiculture* (ed. Satchell JE) (Chapman & Hall, London, 1983).

31	Whitaker J. O. , Hamilton W. J. *Mammals of the Eastern United States* (Cornell University Press, Ithaca NY, 1998).

32	Paradiso, J. L. , Nowak, R. M. Wolves In: *Wild Mammals of North America* Chapman, J. A. , Feldhamer G. A. (Johns Hopkins University Press, Baltimore, 1982)

33	Fagerstone, K. A. Black-footed ferret, longtailed weasel, short-tailed weasel, and least weasel In: *Wild Furbearer Management and Conservation in North America.* Novak, M. , Baker, J. A. , Obbard, M. E. , Mallock, B. (Ontario Trappers Association under auspices of the Ontario Ministry of Natural Resources, Ontario, 1987).

34	Wrogemann, N. *Cheetah Under the Sun* (McGraw-Hill Book Company, Johannesburg, 1975).

35	Avenant, N. L. , Nel, J. A. J.	Home-range use, activity, and density of caracal in relation to prey density*. African Journal of Ecology* **36**, 347-359 (1998).

36	Estes, R. D. *The Behaviour Guide to African Mammals* (The University of California Press, Berkeley, 1995).

37	MacDonald, D. W. , Atkinson, R. P. D. , Blanchard, G. Spatial and temporal patterns in the activity of European moles. *Oecologia* **109**, 88-97 (1997).

38	Seidensticker, J. C. , Hornocker, M. G. , Wiles, W. V. , Messick, J. P. *Mountain Lion Social Organization in the Idaho Primitive Area* (Wildlife Society, Washington D. C. , 1973).

39	Stirling, I. , Oritsland, N. A. Relationships between estimates of ringed seal (Phoca hispida) and polar bear (Ursus maritimus) populations in the Canadian Arctic. *Canadian Journal of Fisheries and Aquatic Sciences* **52**, 2594-2612 (1995).

40	Odonoghue, M. , Boutin, S. , Krebs, C. J. , Murray, D. L. , Hofer, E. J. Functional responses of coyotes and lynx to the snowshoe hare cycle. *Ecology* **79**, 1193-1208 (1998).

41	Gorman, M. L., Mills, M.G., Raath, J. P. & Speakman, J. R. High hunting costs make African wild dog vulnerable to kleptoparasitism by hyaenas. *Nature* **391**, 479-481 (1998).

42	Nagy, K. A., Girard, I. A. & Brown, T. K. Energetics of free-ranging mammals, reptiles, and birds. *Annual Review of Nutrition* **19**, 247-277 (1999).

43	Williams, J. B., Lenain, D., Ostrowski, S., Tieleman, B. I. & Seddon, P. J. Energy expenditure and water flux of Ruppell's foxes in Saudi Arabia. *Physiological and Biochemical Zoology* **75**, 479-488 (2002).

44	Aldama, J. J., Beltran, J. F. & Delibes, M. Energy expenditure and prey requirements of free-ranging Iberian lynx in southwestern Spain. *J. Wildlife Management* **55**, 635-641 (1991).

45	Laundre, J. W. & Hernandez, L. Total energy budget and prey requirements of free-ranging coyotes in the Great Basin desert of the western United States. *Journal of Arid Environments* **55**, 675-689 (2003).

46	Powel, R.A. Ecological Energetics and Foraging Strategies of the Fisher (Martes pennanti), *Journal of Animal Ecology* **48**, 195-212 (1979).
